# Dual-Channel Probe of Carbon Dots Cooperating with Lanthanide Complex Employed for Simultaneously Distinguishing and Sequentially Detecting Tetracycline and Oxytetracycline

**DOI:** 10.3390/nano12010128

**Published:** 2021-12-31

**Authors:** Lei Jia, Zhitao Xu, Rujie Chen, Xiangzhen Chen, Jun Xu

**Affiliations:** College of Chemistry and Chemical Engineering, Henan Polytechnic University, Jiaozuo 454000, China; jlxj@hpu.edu.cn (L.J.); xzhthpu@163.com (Z.X.); chenrujiehpu@163.com (R.C.); cxzhpu@163.com (X.C.)

**Keywords:** molecularly imprinted route, rare earth, carbon dots, distinguishing detection, tetracyclines

## Abstract

Tetracycline (TC) and oxytetracycline (OTC) are the most widely used broad-spectrum antimicrobial agents in tetracycline drugs, and their structures and properties are very similar, so it is a great challenge to distinguish and detect these two antibiotics with a single probe at the same time. Herein, a dual-channel fluorescence probe (SiCDs@mMIPs-cit-Eu) was developed by integrating two independent reaction sites with SiCDs-doped mesoporous silica molecular imprinting group and europium complex group into a nanomaterial. The synergistic influence of inner filter effect and “antenna effect” can be guaranteed to solve the distinction between TC and OTC. Moreover, this novel strategy can also sequentially detect TC and OTC in buffer solution and real samples with high sensitivity and selectivity. This method revealed good responses to TC and OTC ranging from 0 to 5.5 μM with a detection limit of 5 and 16 nM, respectively. Combined with the smartphone color-scanning application, the portable and cheap paper-based sensor was designed to realize the multi-color visual on-site detection of TC and OTC. In addition, the logic gate device was constructed according to the fluorescence color change of the probe for TC and OTC, which provided the application possibility for the intelligent detection of the probe.

## 1. Introduction

Tetracyclines (TCs) are a widely used tetracycline antibiotic, which are often used to treat human and animal infections caused by both Gram-positive and Gram-negative bacteria [1,2]. Because of their remarkable antibacterial effect, TCs are often used as a feed additive to prevent and treat diseases and promote the growth and development of animals in the process of animal breeding [3]. However, the unreasonable use of TCs, including tetracycline (TC) and oxytetracycline (OTC), may lead to their accumulation in the human body because of the residues in animal derived foods, such as milk, eggs, meat, and honey [4,5,6,7]. Although antibiotics have helped humans conquer many diseases caused by bacteria, more than 10% of people are also suffering from the side effects of antibiotics [8]. The study found that tetracycline antibiotics can kill beneficial microorganisms, directly cause the proliferation of drug-resistant bacteria [9,10]. Along with controlling pollution sources and reducing emissions, the simultaneous and rapid detection of multiple pollutants in complex environments can not only effectively reflect the environmental toxicology status and pollutant effects in real time, but also provide technical support for pollution source control, environmental planning and management, and public health and safety protection. However, the half-lifetimes of different kinds of tetracyclines (such as tetracycline and oxytetracycline) are diverse in the animal tissues. It is very conducive to develop effective pollution control strategies and early treatment options by distinguishing them from each other and detecting them. Hence, various methods, such as high-performance liquid chromatography [11], colorimetric analysis [12], capillary electrophoresis [13], and enzyme-linked immunosorbent assay [14] are designed to monitor TCs. However, the above traditional detection methods have some disadvantages, such as expensive equipment, low sensitivity, difficult real-time detection, and so on. Therefore, it is necessary to establish a low-cost, simple, sensitive, stable, and reliable method for the detection of tetracycline residues in food. 

With the continuous development and improvement of detection techniques and methods, various analytical detection methods have been derived through the fluorescence changes between fluorescent groups and specific analytes [15,16,17]. Many TCs fluorescence probes based on enhanced or weakened fluorescence signals have been designed [18,19]. However, it is difficult to distinguish and detect TC and OTC in real time due to their similar structure and chemical properties [20]. Therefore, the development of a simple fluorescence method to replace large-scale instrumental analysis methods is very meaningful. 

It is known that traditional organic dye molecules [21] or quantum dots (QDs) [22] can be used as fluorescent probes, but organic dyes have great toxicity to the human body or the environment, and QDs often contain toxic metal elements, so their biological safety is poor. Carbon dots (CDs), a class of “zero-dimensional” carbon materials, which are obtained at high temperature or microwave reaction, in which most of the constituent elements are chemically inert carbon. And even if the particle size is generally small, CDs still have good biological safety [23]. Due to their unique properties consisting of excellent fluorescent properties such as broad excitation spectra, narrow and tunable emission spectra, and high photostability, CDs have aroused great interest as functional fluorescent probes in the field of biosensing and pollutant detection [24,25,26]. However, fluorescent probes based on carbon dots usually have only one fluorescence, which can only be detected by a single fluorescence enhancement or fluorescence weakening. Due to its single color change, the visual detection sensitivity is low compared with those probes with various fluorescence colors [27,28,29,30]. Therefore, a CDs-based probe should be modified by appropriate methods to solve the above two defects. On the one hand, chemists have realized the study of molecular recognition using some natural or synthetic compounds to simulate biological systems. The molecularly imprinted polymers (MIPs) prepared by this molecular imprinting technique have specific recognition and selective adsorption properties, which are simple and low-cost choices for improving fluorescence detection selectivity [31,32,33]. On the other hand, the construction of dual-channel ratio fluorescence probe, may effectively avoid the problems of low sensitivity and susceptibility to external environment interference from the on-off fluorescence switch sensor, and realize rapid visual detection through the change of fluorescence colors [34,35]. 

**Scheme 1:**
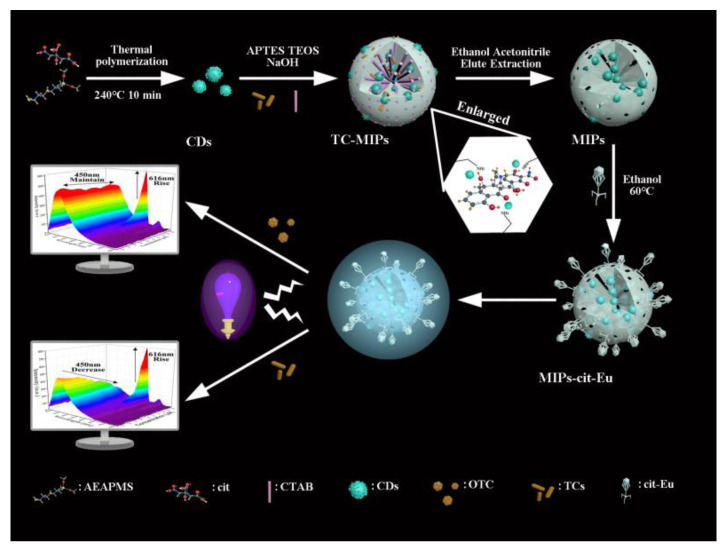
The schematic diagram of the preparation of SiCDs@mMIPs-cit-Eu nanoprobe and distinction and detection TC and OTC.

Herein, we described a novel discrimination strategy for TC and OTC based on two potential recognition sites of a europium functionalized organosilane-functionalized carbon dots (SiCDs) doped mesoporous silica molecularly imprinted probe (Scheme 1). The probe could not only distinguish TC or OTC from each other but also simultaneously recognize TC and OTC using different fluorescent emission channels and thus hold great potential in food safety. The design principle was described in Scheme 1 and illustrated as follows. The blue emission channel was composed of mesoporous silica molecularly imprinted SiCDs. The template molecule TC can quench the fluorescence of SiCDs due to inner filter effect (IFE) fluorescence quenching mechanism, while OTC can hardly quench the fluorescence of SiCDs. The red emission channel was composed of citric acid chelated europium complex, which was grafted on the surface of mesoporous silica. Due to TC and OTC containing β-diketone configuration, they can coordinate and transfer energy to Eu^3+^ and sensitize the Eu^3+^ emission through the “antenna effect” [36,37]. Because the triplet energy levels of TC and OTC were different, their energy transfer to Eu^3+^ ions were also different. It would also be possible to distinguish TC and OTC by the fluorescence changes in red emission channels. Taken together, the differences in the structure and properties of TC and OTC could change the interaction with SiCDs and europium complexes, so that they can be distinguished from each other and detected through dual-channel fluorescent emission. Meanwhile, combined with the smart phone, a portable and cheap paper-based sensor was designed to realize the multi-color visual detection of the antibiotics. In addition, the logic gate device was constructed according to the fluorescence color change of the probe for TC and OTC, which provided the application possibility for the intelligent detection of the probe.

## 2. Materials and Methods 

### 2.1. Materials and Reagents

N-(b-aminoethyl)-g-aminopropylmethyl dimethoxysilane (AEAPMS), europium nitrate (Eu(NO_3_)_3_·6H_2_O, 99.99%), tetraethyl orthosilicate (TEOS), 3-aminopropyltriethoxysilane (APTES), citric acid anhydrous(cit), and hexadecyl trimethyl ammonium bromide (CTAB) were purchased from Aladdin Reagent Co., Ltd. (Shanghai, China). N, N-dimethylformamide (DMF), hydrochloric acid (HCl), and ethanol were purchased from Kermel analytical reagent Co., Ltd. (Tianjing, China). 1-ethyl-3-(3-dimethylaminopropyl) carbodiimidehy -drochloride (EDC), N-hydroxysuccinimide (NHS), Dicyclohexylcarbodiimide (DCC), and tris(hydroxymethyl)aminomethane (Tris) were purchased from Acros Organics. Tetracycline (TC) was purchased from Xiya reagent Co., Ltd. (Shandong, China). Oxytetracycline (OTC), thiamphenicol, kanamycin sulfate, ofloxacin, chloramphenicol, and potassium bromide (KBr, spectral pure) were purchased from Adamas-beta Reagent Co., Ltd. (Shanghai, China). Potassium chloride (KCl), sodium chloride (NaCl), calcium chloride (CaCl_2_), magnesium chloride (MgCl_2_), ferric chloride hexahydrate (FeCl_3_·6H_2_O), sodium nitrate (NaNO_3_), potassium nitrate (KNO_3_), potassium sulfate (K_2_SO_4_), sodium sulfate (Na_2_SO_4_), and sodium hydroxide (NaOH) were purchased from Shanghai Titan Scientific Co., Ltd. (Shanghai, China). All reagents are of analytical grade or higher and used without further treatment.

### 2.2. Synthesis of the SiCDs

The organosilane-functionalized carbon dots (SiCDs) were synthesized from anhydrous citric acid using methods which already reported in the literature [38]. Firstly, 20 mL of AEAPMS in a three-neck bottle was heated to 240 °C under nitrogen atmosphere, and 0.5 g of anhydrous citric acid powder was quickly added to the above system. The resulting mixed solution was reacted at 240 °C for 10 min. The resulting solid was centrifuged and washed with petroleum ether. The obtained solid were dispersed in ultrapure water and the large particles were removed through a 0.22 μm membrane. The filtered clear liquid was further dialysis using a dialysis bag (1000 Da). Finally, the clear liquid after dialysis was vacuum freeze-dried to obtain CDs powder.

### 2.3. Synthesis of Mesoporous SiCDs@mMIPs

Molecularly imprinted polymers embed with carbon quantum dots (SiCDs@mMIPs) was prepared by a conventional sol-gel method as follows. A total amount of TC (5 mg), CDs (10 mg), and CTAB (200 mg) were dispersed in 50 mL of ultrapure water, followed by the injection of APTES (1000 μL) and TEOS (250 μL) into the resulting mixture with stirring. After that, 0.75 mL of ammonium hydroxide (NH_3_·H_2_O, 6% *v/v*) was introduced to the flask. Then the reaction was protected from light under mechanical agitation for 12 h. The product was separated simply by centrifugation after the reaction and the TC template molecules in the imprinted layer were eluted with acidified methanol (95:5, *v/v*) at least 8 times. Control experiments were also carried out following the same procedure in the absence of the TCs template. The no-imprinted polymers (NIPs) were synthesized according to the same steps above without template (tetracycline).

### 2.4. Synthesis of SiCDs@mMIPs-APTES

A mass of 100 mg of SiCDs@mMIPs powder was dispersed in the mixed solution (ethanol:water = 1:1, 30 mL) by ultrasonic treatment for 1 h. Following the filtration through a 0.22-μm membrane, 500 μL of APTES was added to reaction system with vigorous stirring for 30 min, finally 1 mL NH_3_·H_2_O was added and stirred overnight. After the reaction was completed, the white solid was collected and washed three times with anhydrous ethanol, the final product was dried at 60 °C.

### 2.5. Synthesis of SiCDs@mMIPs-cit

A mass of 50 mg of anhydrous citric acid was dispersed in the mixed solution (DMF:water = 1:1, 20 mL) and ultrasonic treatment for 5 min. Following that, 10 mg of EDC, 10 mg of NHS, and 10 mg of DCC were sequentially added to the reaction system and stirred vigorously for 4 h at room temperature. After adding the SiCDs@mMIPs-APTES and stirring for 24 h, the resulting SiCDs@mMIPs-cit complex was collected and dried for further modification.

### 2.6. Synthesis of SiCDs@mMIPs-cit-Eu

A mass of 50 mg of SiCDs@mMIPs-cit powder was dispersed in 15 mL of ethanol and ultrasonic treatment for 1 h, then 15 mg of Eu(NO_3_)_3_·6H_2_O was added into the solution and reacted at 60 °C in an oil bath for 8 h. Finally, the product was centrifuged and washed with ultrapure water three times.

### 2.7. Analytical Procedure

To measure the detection effect of the probe, 100 μL of stock solution of SiCDs@mMIPs-cit-Eu (1 mg/mL) was added to a 4.0 mL standard cuvette and the final volume of the mixture was maintained at 2 mL by the addition of Tris-HCl buffer (pH = 8). Different concentrations of TC or OTC (in the range of 0–10 μM) in aqueous solution were added to the above solution and reacted for 5 min at room temperature, the ratios of fluorescence intensities at 616 and 450 nm (I_616_/I_450_) were measured using excitation wavelength of 360 nm.

### 2.8. Preparation of Test Paper-Based Ratiometric Fluorescent Sensor Surpported by Smartphone

The paper sensor was modified by ordinary circular neutral qualitative filter paper. The filter paper was soaked with SiCDs@mMIPs-cit-Eu dispersion (1 mg/mL) for 5 min, then dried for further use. During the test, 5 μL of TC or OTC solutions of different concentrations were added to the surface of the above filter paper, and fluorescent photos of various colors were collected under UV light. The color ratio (R/B) was calculated by the chromaticity extraction and analysis program in the smartphone, and the rapid visual detection of different tetracyclines was realized.

### 2.9. Characterization

The instruments used for material characterization are transmission electron microscope (TEM) (JEM-2100, Jeol, Japan) and Fourier transform infrared (FT-IR) spectrometer (FI-IR, VERTEX 70, Bruker, Billerica, MA, USA). The luminescence spectra were measured on a Varian CARY Eclipse fluorescence spectrometer. UV-vis absorption spectra were determined on a Varian UV-Cary100 spectrophotometer. Ultrapure water was prepared by using Reverse Osmosis Technology Specialist (Aquapro, ABY-1002-U, Hamilton, OH, USA). X-ray photoelectron spectra (XPS) were determined by X-ray photoelectron spectrometer (ThermoFisher, ESCALAB 250Xi) using monochromatic Al Kα radiation (h*ν* = 1486.6 eV), operating at an accelerating power of 150 W. All the powder samples were compacted into 1 cm × 1 cm sheets before XPS test and sputter-etching performed by Ar^+^ ions was employed. The binding energies in the XPS spectra were calibrated against the adventitious carbon C (1s) singlet (284.84 eV).

## 3. Results and Discussion

### 3.1. Preparation and Characterization of SiCDs

The SiCDs were synthesized by the previously reported procedures include the thermal decomposition of anhydrous citric acid and passivation of amino group in silane coupling agent with carboxyl group in cit, which was further confirmed by FTIR analysis. The peaks of 1650 and 1564 cm^−1^ in Figure S1a can be assigned to the vibrations of amide I and amide II, respectively, indicated the presence of -CO–NH groups [39], and -NH_2_ [40] in the SiCDs. Organosilicon can introduce CDs into porous silica particles in the process of hydrolysis and condensation, which can facilitate the synthesis of molecular imprinting layer. As shown in Figure S1b, the peak of 1060 cm^−1^ was oriented from asymmetric stretching vibration of Si-O-Si group, and the peaks at 787 and 457 cm^−1^ could be assigned to the bending vibration of an Si-O group, demonstrating that the molecular imprinting layer were successfully formed on the surface of the SiCDs [41]. The template molecule TC was extracted by traditional solvent elution method. The peaks at 1494 and 1450 cm^−1^ (Figure S1b) can be ascribed to the aromatic ring stretching vibrations of TC template molecules, which disappeared after TC extraction (Figure S1c). The above results showed that TC compounds can not only be imprinted into porous silica, but also can be separated from the matrix by solvent elution, which was confirmed by UV-vis absorption spectra. Figure S2 indicated that after washing five times with acidified methanol, the characteristic peak TC disappeared, demonstrating the effective removal of TC from SiCDs@MIP.

**Figure 1:**
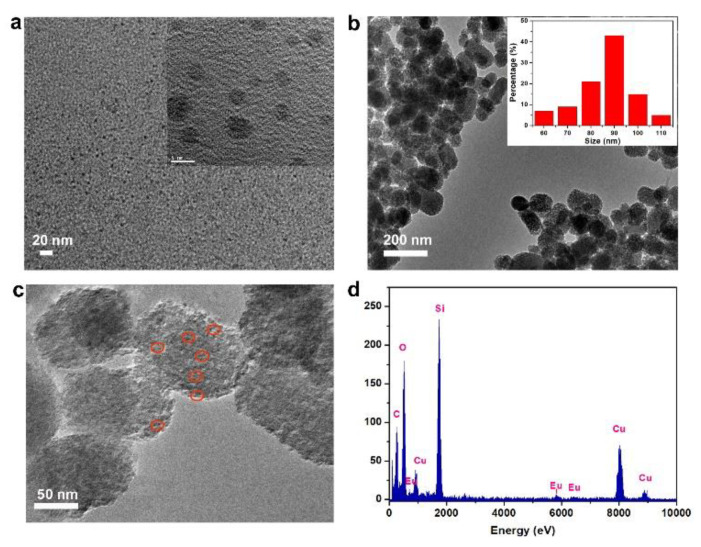
TEM images of SiCDs (**a**) and SiCDs@mMIP-cit-Eu probe (**b**). Inset: HRTEM image of SiCDs and size distribution image of SiCDs@mMIP-cit-Eu. (**c**) HRTEM image and (**d**) EDS spectra of SiCDs@mMIP-cit-Eu probe.

In order to confirm the successful preparation of this dual-channel probe, the morphologies of SiCDs and SiCDs@mMIP-cit-Eu were investigated by TEM (Figure 1). As shown in Figure 1a, SiCDs had a spherical shape with an average diameter of about 4 nm, while the nanoparticles had no obvious lattice, indicating that the particles are amorphous. As illustrated in the XRD pattern (Figure S3), a broad peak at around 23° assigned to the (002) diffraction planes of carbon materials can further confirm an amorphous structure in SiCDs. After the extraction of template molecule (TC) and pore-forming agent (CTAB), the mesoporous molecular imprinting silica layer was formed, and the amorphous structure of SiCDs remained (Figure S3). The average diameter of the SiCDs@mMIP-cit-Eu nanoprobe was about 90 nm (inset of Figure 1b). The mesoporous structure in silica shell doped with SiCDs in SiCDs@mMIP-cit-Eu was obvious in the HRTEM image (Figure 1c), which was expected to increase the contact probability between the target molecule and the probe [42]. Moreover, in addition to the signal from the Cu in the TEM grid, the energy dispersive X-ray spectra (EDS, Figure 1d) demonstrated the presence of C, O, Si, and Eu elements in the nanoprobe, indicating the successful grafting of cit-Eu complex.

**Figure 2:**
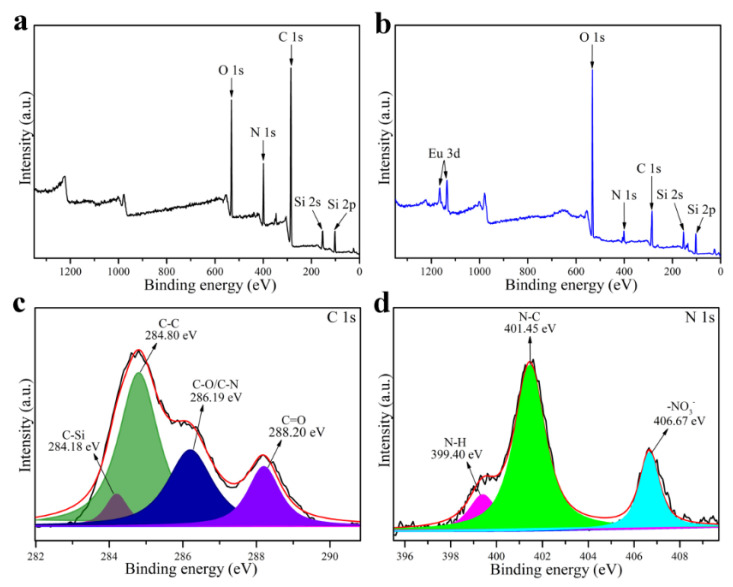
X-ray photoelectron spectra of SiCDs (**a**) and SiCDs@mMIP-cit-Eu probe (**b**). The detailed C 1s (**c**) and N 1s (**d**) spectra of SiCDs@mMIP-cit-Eu.

In addition to the above analysis, the elemental composition of SiCDs and SiCDs@mMIP-cit-Eu were further analyzed by XPS technique. As shown in Figure 2a, the XPS full-scan spectrum of SiCDs showed five strong peaks at 102.2, 153.4, 285.0, 399.0, and 532.0 eV, which can be attributed to Si 2p, Si 2s, C 1s, N 1s, and O 1s, respectively. After molecular imprinting process and further grafting of cit-Eu, the surface composition of SiCDs@mMIP-cit-Eu nanoprobe was also demonstrated by the XPS spectra analysis. There were six peaks in the XPS full-scan spectrum (Figure 2b), indicating that the SiCDs@mMIP-cit-Eu were mainly composed of Si, C, N, O, and Eu. The high resolution XPS of C 1s (Figure 2c) can be divided into to four peaks at 284.2, 284.8, 286.2, and 288.2 eV, demonstrating the existence of C-Si, C-C, C-O/C-N, and C=O bonds on the surface of nanoprobe [43,44]. The N 1s spectrum in Figure 2d can be deconvoluted into three peaks, ascribed to N−H at 399.4 eV, N−C at 401.5 eV, and NO_3_^−^ at 406.6 eV, respectively [45,46]. These results supported that cit-Eu complex had been successfully grafted on the surface of SiCDs@mMIP.

**Figure 3:**
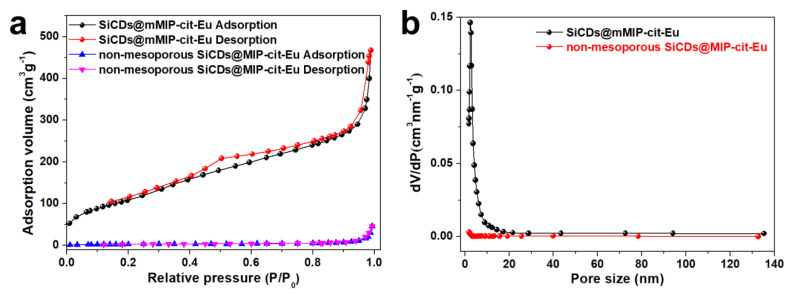
(**a**) The nitrogen adsorption–desorption isotherms of SiCDs@mMIP-cit-Eu and non-mesoporous SiCDs@MIP-cit-Eu materials. (**b**) Pore size distributions of iCDs@mMIP-cit-Eu and non-mesoporous SiCDs@MIP-cit-Eu materials.

In addition, the mesoporous structure of SiCDs@mMIP-cit-Eu was further confirmed by nitrogen adsorption–desorption and pore size distributions experiment (Figure 3). The relative pressure in the typical IV-type isotherm curve (Figure 3a) increased rapidly from 0.4 to 0.5, indicating that there are uniform mesopores in SiCDs@mMIP-cit-Eu [47]. The average pore size obtained from the adsorption isotherm was about 2.5 nm. The mesoporous property of SiCDs@mMIP-cit-Eu was further proved by BET analysis (Figure 3b). The specific surface area of SiCDs@mMIP-cit-Eu was 404.4 m^2^·g^−1^, which was much higher than the specific surface area of the corresponding non-mesoporous nanomaterial (11.7 m^2^·g^−1^). This structure may be beneficial to improve the selective recognition ability and sensitivity of TC. 

### 3.2. Optimal Detection Conditions of SiCDs@mMIP-cit-Eu Nanoprobe for TC

In order to optimize the detection conditions, the optimal response time and reaction pH of as-prepared nanoprobe were investigated. The fluorescence of Eu^3+^ was dramatically quenched when pH was less than 6.0 or more than 11.0 (Figure S4a). This may because the acidic condition inhibited the coordination of TC with Eu^3+^, while under alkaline condition, Eu(III) existed in the form of stable hydroxide, which was difficult to be affected by the concentration of TC. The fluorescence intensity of SiCDs maintained in the range of pH from 4.0 to 9.0 (Figure S4b). Therefore, the subsequent tests were carried out in the buffer solution of pH = 8.0. The selection of fluorescence response time had an important influence on the performance of the probe. Figure S5a indicated the fluorescence intensity of SiCDs decreased continuously with the change of time, which became stable after the reaction of 5.0 min. From Figure S5b, it can be found that the fluorescence intensity of Eu^3+^ obviously enhanced after adding TC, and the fluorescence tended to be stable after 5 min, so the optimal response time of the probe was determined to be 5 min. We also studied the stability of SiCDs and SiCDs@mMIPs-cit-Eu under high-voltage illumination of xenon lamp for 800 s (as shown in Figure S6). The results showed that the fluorescence intensities at 450 and 616 nm were stable, which can meet the requirement of rapid detection of TC.

### 3.3. Sensing and Distinguishing Mechanism

**Figure 4:**
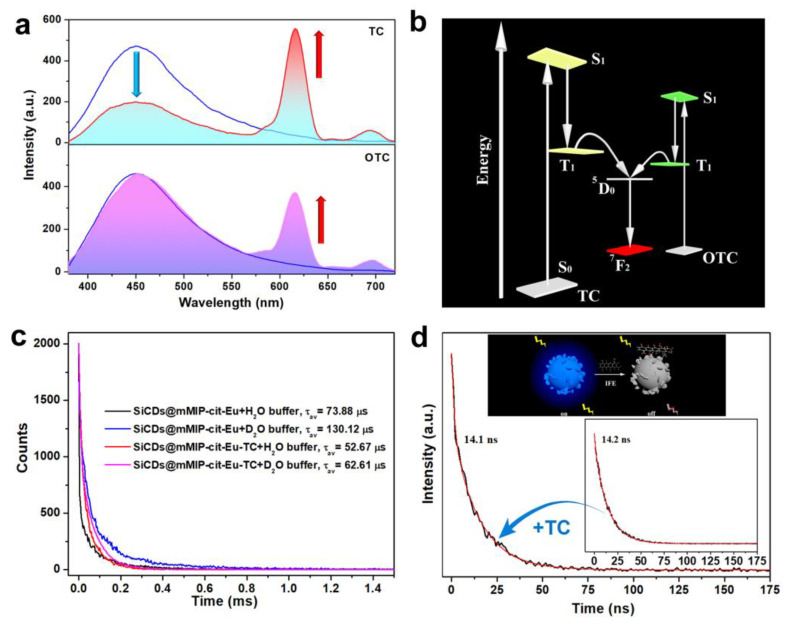
(**a**) Fluorescence emission spectra of SiCDs@mMIP-cit-Eu in the absence and presence of 3.5 μM of TC (top) and OTC (bottom). (**b**) Energy transfer mechanism diagram from TC and OTC to Eu^3+^. (**c**) The lifetime of SiCDs@mMIP-cit-Eu and SiCDs@mMIP-cit-Eu-TC nanomaterials in H_2_O and D_2_O buffers. (**d**) The lifetime SiCDs in the absence (right) and presence (left) of TC (Inset: schematic diagram of IFE process).

The detection process and sensing mechanism of SiCDs@mMIP-cit-Eu were illustrated in Figure 4. At first, SiCDs@mMIP-cit-Eu nanoprobe can emit strong blue fluorescence at 450 nm of SiCDs and weak red luminescence of Eu^3+^ (dominated at 616 nm). After adding a certain concentration of TC or OTC, the fluorescence intensity of Eu^3+^ increased, and the characteristic emission bands of Eu^3+^ at 591, 616, and 697 nm were obvious (Figure 4a). The fluorescence enhancement mechanism can be attributed to two reasons. Firstly, the β-diketone configuration of both TC and OTC can strongly chelate with Eu^3+^ ions through metal–ligand interaction and effectively transfer energy to Eu^3+^ ions via “antenna effect”, and the energy transfer process usually began with the lowest triplet (T_1_) of the ligand donor and followed the Dexter exchange principle. As shown in Figure 4b, the T_1_ state of TC and OTC were 19,500 and 19,160 cm^−1^ [48], which were suitable for the energy transfer from the ligand to the central Eu^3+^ ions (^5^D_0_ level, 17,300 cm^−1^), thus sensitizing the fluorescence of Eu^3+^ ions. Secondly, the coordinated water molecule occupied the first coordination layer in the binary complex (cit-Eu), due to the coordination of the central metal Eu ion was unsaturated. The strong chelation of TC and OTC with Eu^3+^ ions can replace the coordinated water molecules and reduce the vibronic coupling with O-H oscillator from water molecules, resulting in the enhancement of Eu(III) emission. This statement can be verified by measuring the number of coordinated water molecules in the absence and presence of TC and OTC. The numbers of coordinated water molecules (n_w_) in SiCDs@mMIP-cit-Eu, SiCDs@mMIP-cit-Eu-TC, and SiCDs@mMIP-cit-Eu-OTC can be determined according to the equation developed by Horrocks and Supkowski [49]:

n_w_ = 1.1[τ_H2O_^−1^ − τ_D2O_^−1^ − 0.31] (1)

where τ_H2O_ and τ_D2O_ are the lifetime of nanomaterials in H_2_O and D_2_O buffers, respectively. The calculated results from the fluorescence lifetime (Figure 4c and Figure S7) showed that n_w_ decreased from 6.1 in the SiCDs@mMIP-cit-Eu to 2.9 and 3.1 in the SiCDs@mMIP-cit-Eu-TC and SiCDs@mMIP-cit-Eu-OTC, respectively. Therefore, the binding of TC can shield the coordinated water molecules effectively and prevent the fluorescence quenching caused by the vibrational coupling with the O-H oscillator.

In the presence of the imprinted molecule TC, the blue fluorescence emission of the SiCDs was quenched, while the non-imprinted molecule OTC had no effect on the blue emission channel. Generally, there are three common mechanisms of fluorescence quenching, including inner filter effect (IFE), fluorescence resonance energy transfer (FRET), and static quenching [31,32,50]. In order to study the fluorescence quenching mechanism at 450 nm, the UV spectra, fluorescence spectra and the fluorescence lifetime in the absence and presence of tetracycline were investigated. Firstly, the static quenching was excluded by the analysis of UV spectra. The UV-Vis absorption spectra of SiCDs, TC, and the mixture of SiCDs and TC (Figure S8) indicated that there was no additional absorption bond, and the position of the absorption peak was hardly changed, indicating that there was no new substance formation [45]. Moreover, the fluorescence emission of SiCDs overlapped with the absorption spectrum of TC, and it can be speculated that the fluorescence quenching may be caused by IFE or FRET [32].

FRET is an electrodynamic phenomenon that can be explained by using classical physics. The fluorescence lifetime of SiCDs would decrease and SiCDs-quencher distance would be in the range of 10–100 Å can demonstrate that the mechanism for SiCDs-quenching was FRET [50]. However, the fluorescence lifetime of SiCDs (14.2 ns) was almost unchanged after adding TC (14.1 ns), which could be speculated that the fluorescence of SiCDs was greatly quenched by template molecule TC via IFE instead of FRET [50,51].

### 3.4. Sensitive Performance of SiCDs@mMIP-cit-Eu for the Detection of TC and OTC 

**Figure 5:**
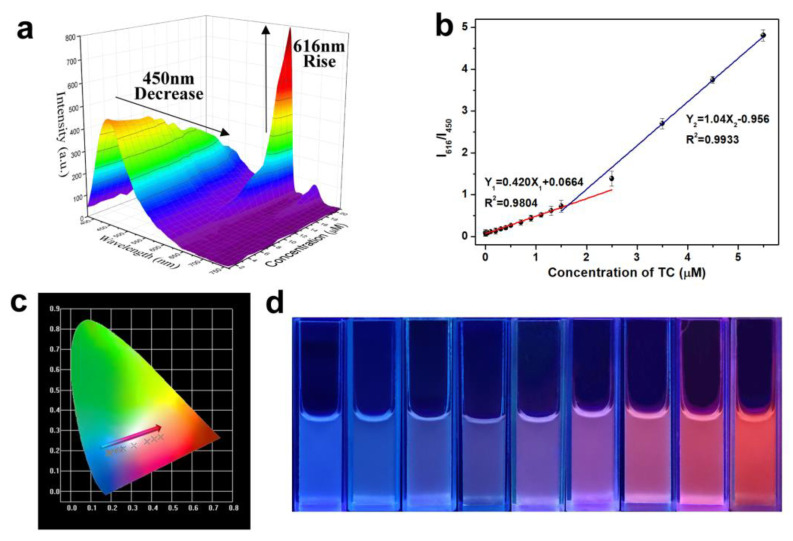
(**a**) The emission spectra of SiCDs@mMIP-cit-Eu in the presence of different TC concentrations. (**b**) The fitting curve of SiCDs@mMIP-cit-Eu between the relative ratios of (I_616_/I_450_) and TC concentrations ranging from 0 to 5.5 μM, and the error bars represent the standard deviation from three parallel tests. (**c**) CIE chromaticity diagram showing the (x,y) fluorescent color coordinates of SiCDs@mMIP-cit-Eu with the TC concentrations ranging from 0 to 5.5 μM. (**d**) The fluorescence images of SiCDs@mMIP-cit-Eu probe in the presence of 0, 0.1, 0.5, 1, 2, 2.5, 5, 10, and 20 μM TC under a UV lamp at 365 nm.

In order to investigate the good sensitive performance of SiCDs@mMIP-cit-Eu for the detection of TC and OTC, fluorescence titration and visual fluorescence imaging experiment were carried out (Figure 5). As shown in Figure 5a, the fluorescence intensity of SiCDs@mMIP-cit-Eu at 616 nm increased obviously after adding various amounts of TC, while the fluorescence intensity at 450 nm decreased gradually. The ratio (I_616_/I_450_) of the fluorescence intensity was closely related to the concentrations of TC. There were two linear relationship between the fluorescence ratio I_616_/I_450_ and the concentrations of TC in the range from 10 nM to 1.5 μM and from 1.5 to 5.5 μM (Figure 5b), and the detection limit was about 5 nM based on the ratio of signal-to-noise of 3. The Commission Internationale de l’Eclairage’s (CIE) chromaticity diagram showed the change of fluorescence emission of SiCDs@mMIP-cit-Eu at different TC concentrations (Figure 5c) [16], and the corresponding chromaticity coordinates were also provided in Table S1. Under the UV lamp, it can be clearly observed that with the gradual increase of TC content, the fluorescence color of the detection system showed a gradual change from blue to red (Figure 5d). The detection performances of different kinds of TC probes reported in the literature were listed in Table S2. The detection performance of this probe was superior to or at least comparable to several reported TC detection methods in terms of linear range and detection limit. Moreover, other methods usually required preprocessing, which were time consuming and expensive [11,13,14], and this method can achieve rapid visual detection. 

**Figure 6:**
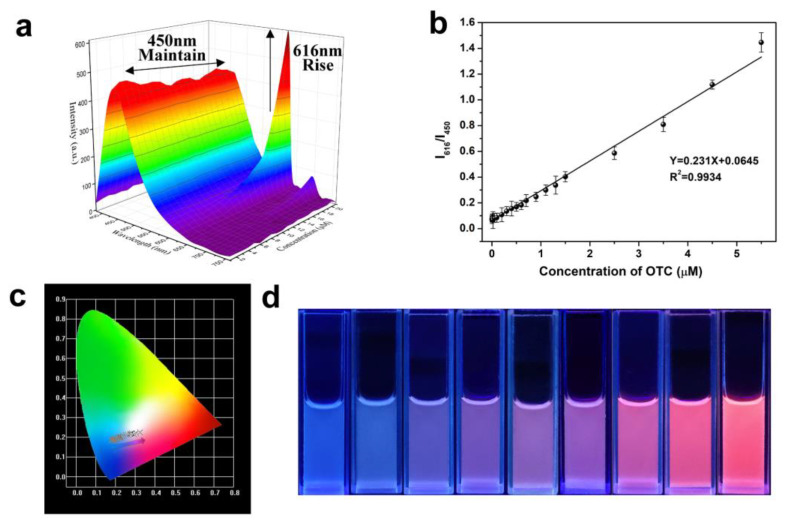
(**a**) The emission spectra of SiCDs@mMIP-cit-Eu in the presence of different OTC concentrations. (**b**) The fitting curve of SiCDs@mMIP-cit-Eu between the relative ratios of (I_616_/I_450_) and OTC concentrations ranging from 0 to 5.5 μM, and the error bars represent the standard deviation from three parallel tests. (**c**) CIE chromaticity diagram showing the (x,y) fluorescent color coordinates of SiCDs@mMIP-cit-Eu with the OTC concentrations ranging from 0 to 5.5 μM. (**d**) The fluorescence images of SiCDs@mMIP-cit-Eu probe in the presence of 0, 0.1, 0.5, 1, 2, 2.5, 5, 10, and 20 μM OTC under a UV lamp at 365 nm.

More interestingly, as shown in Figure 5, the probe can not only realize the rapid visual detection of TC, but can also distinguish OTC from TC through different fluorescent emission channels. The Eu^3+^ fluorescence intensity at 616 nm of SiCDs@mMIP-cit-Eu increased with the increasing amounts of OTC (Figure 6a), whereas the blue fluorescence of SiCDs remained constant. The different responses of two emission channels can realize simultaneous differential detection of TC and OTC. Similarly, the ratio (I_616_/I_450_) of the fluorescence intensity increased when the content of OTC ranged from 0 to 5.5 μM, and the detection limit was 16 nM (Figure 6b). At the same time, from the CIE color coordinate diagram (Figure 6c) and the naked eye observation image (Figure 6d) under the ultraviolet lamp, we can also see that the transition color of the probe for OTC fluorescence sensing was different from that of TC fluorescence sensing. The detection performances of different kinds of OTC probes reported in the literature were listed in Table S3. The detection performance of SiCDs@mMIP-cit-Eu nanoprobe was superior to or at least comparable to several reported TC detection methods in terms of linear range and detection limit.

### 3.5. Interference of Coexisting Substance

In order to determine the effects of different external environments on the determination of TC and OTC, the fluorescence changes in the presence of many interfering coexisting substances such as Cys, His, Lys, GSH and ascorbic, metal cations (Na^+^, K^+^, Ca^2+^, Fe^3+^, and Mg^2+^), anions (NO_3_^-^ and Cl^-^), and similar species (thiamphenicol (TAP), kanamycin sulfate (KS), ofloxacin (OFL), adriamycin hydrochloride (ADMh), tigecycline (TIC), minocycline (MCh), and chloramphenicol (CAP)) were studied. As shown in Figures S9 and S10, no significant fluorescence intensity or the peak position changes in SiCDs@mMIP-cit-Eu-TC can be observed after introducing the coexisting substances, suggesting that the probe had good selective recognition ability and can be expected to be used in the detection of TC in a variety of complex environments.

### 3.6. Detection of TC in Real Samples

It is of great significance to apply SiCDs@mMIP-cit-Eu nanoprobe to the analysis of TC in real samples for food safety and ecological environment monitoring. The pretreated milk [52,53], honey, tap water, and river water, were selected as the real samples. As listed in Table S4, when the concentration of TC was 0, 0.1, 1, and 5 μM, respectively, the recoveries were in the range of 96.5–104.7% and relative standard deviations (RSD, *n* = 3) was below 3%. These results indicated that the SiCDs@mMIP-cit-Eu probe could assay TC and OTC with good accuracy and reliability.

### 3.7. Smartphone and Test Paper-Based Ratiometric Fluorescent Sensor for Visual Detection of TC

**Figure 7:**
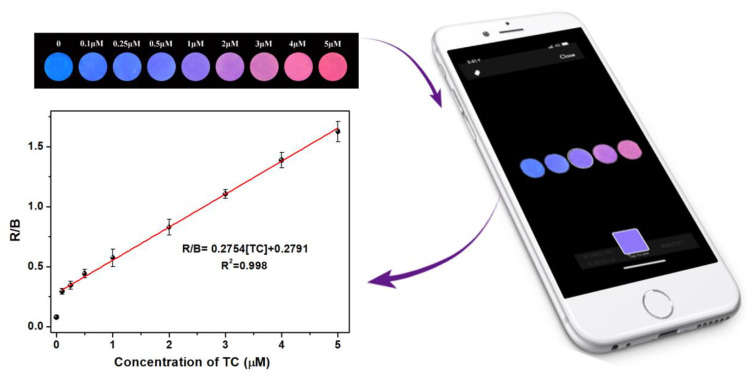
Fluorescence color images the SiCDs@mMIP-cit-Eu immobilized test paper at the different TC concentrations under a 365 nm UV lamp, and the corresponding relationship between TC concentrations and color ratio (R/B) achieved by a smartphone color-scanning APP.

Due to the advantages such as high sensitivity, selectivity, and rapid response, a portable SiCDs@mMIP-cit-Eu immobilized filter paper-based sensor was developed for on-site TC detection (Figure 7). With the continuous increase of TC content, the fluorescent color of the SiCDs@mMIP-cit-Eu immobilized test paper can be observed from blue to red under the 365 nm UV lamp. However, the SiCDs@mMIP-cit-Eu immobilized test paper was difficult to quantify the concentration of TC by the naked eye. For this reason, the semi-quantitative analysis of TC was carried out by using a color-scanning APP (World of Color) downloaded online from a smartphone [16], and the color signals can be converted into color information (RGB value) [54,55]. Since the fluorescent color of the paper-based detection system evolved continuously from blue to red with the increase of TC concentrations, the ratio of red (R) to blue (B) was employed to TC content determination. As shown in Figure 6, the color ratio (R/B) had a good linear correlation with TC concentrations in the range of 0.1–5 μM (R^2^ = 0.998), which also indicated that this smart phone assisted TC semi-quantitative detection platform had great potential to be applied to the on-site detection of actual samples. Furthermore, the lowest detection concentration was 0.1 μM, which was lower than the internationally recognized maximum residue limit set [56]. 

### 3.8. Ratiometric FL Molecular Logic Gate

**Figure 8:**
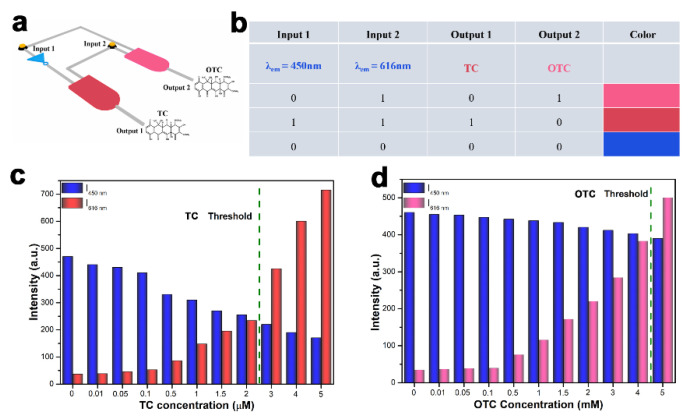
(**a**) Logic circuit based on the corresponding symbols, (**b**) the resulting truth table from different input modes and column diagram of the thresholds for detecting TC (**c**) and OTC (**d**).

The activation and conversion of tetracyclines and different fluorescence signals were further studied by portable UV lamp. According to the significant ratio-FL response of the MIPs in distinguishing TC from OTC, a molecular logic gate was designed. The molecular logic gate used 450 nm fluorescence (Input 1) and 616 nm fluorescence (Input 2) as two inputs, while the type of antibiotics as the outputs, TC was defined as “Output 1” and OTC was defined as “Output2”. Figure 8a,b summarized these corresponding symbols and the resulted truth tables from different input modes. For the output, the presence of antibiotics was defined as the “1” state, and there was no defined/definition as the “0” state. For any input port, the change of the fluorescence signal was defined as “1”, while the constant was defined as “0”. When the blue fluorescence at 450 nm was unchanged, but the red fluorescence at 616 nm was enhanced (0,1), the detected antibiotic was oxytetracycline. When the blue fluorescence at 450 nm was weakened, but the red fluorescence at 616 nm was enhanced (1,1), indicating that the antibiotic was tetracycline. When the two independent fluorescence signals did not change (0,0), it indicated that the tested substance was neither tetracycline nor oxytetracycline. No matter in practice or in theory, it was impossible to have a change in blue fluorescence at 450 nm and no change in red fluorescence at 616 nm (1,0), so this situation was not listed in the truth table. In order to better distinguish whether the color changed or not, the threshold was defined in the interval where the intensity value of I_616_/I_450_ was “1”. As shown in Figure 8c,d, when beyond this interval, the color change in the detection system was obvious enough that antibiotics can be defined or not.

## 4. Conclusions

In brief, a novel strategy for not only simultaneously distinguishing TC and OTC using a dual-channel fluorescence probe was developed. The distinguishing response mechanism can be attributed to the synergistic effect of IFE and “antenna effect”. There were good detection limits at 5 and 16 nM for TC and OTC, respectively, which were better than many other reported methods. The probe showed high selectivity, anti-interference ability and stability, and was applied in actual milk, water, and honey samples. Through the preparation of paper-based sensor and combined with smartphone color-scanning APP, portable visual on-site detection can also be realized. In addition, the logic device was proposed to distinguish TC from OTC based on the difference of fluorescence color, which promoted the further development of detection probe in the field of smart devices. 

## Data Availability

The data presented in this study are available on request from the corresponding author.

## Supplementary Materials

The following are available online at https://www.mdpi.com/article/10.3390/nano12010128/s1, Figure S1: FT-IR spectrum of SiCDs (A), and SiCDs@mMIPs before (B) and after (C) solvent extraction of TC template molecule, Figure S2: UV-vis absorption spectra of TC template molecularly imprinted SiCDs@mMIPs material after 5 times washing with acidified methanol, Figure S3: X-ray diffraction (XRD) patterns of SiCDs, SiCDs@mMIPs and SiCDs@mMIPs-cit-Eu, Figure S4: The effect of pH on fluorescence emission peak at 616 nm (a) and 450 nm (b), Figure S5: The response times of fluorescence emission peaks at 616 nm (a) and 450 nm (b) for TC detection, Figure S6: Fluorescence stability of SiCDs (left) and SiCDs@mMIPs-cit-Eu (right) after adding TC in 800 s, Figure S7: The lifetime of SiCDs@mMIP-cit-Eu and SiCDs@mMIP-cit-Eu-OTC nanomaterials in H_2_O and D_2_O buffers, Figure S8: The excitation and emission spectra of SiCDs, and UV-vis absorption spectra of SiCDs, TC and SiCDs-TC, Figure S9: The influence of coexisting substances on the I_616_/I_450_ of SiCDs@mMIP-cit-Eu in the presence of TC, Figure S10: Fluorescence response (I_616_/I_450_) of SiCDs@mMIP-cit-Eu probe to thiamphenicol (TAP), kanamycin sulfate (KS), ofloxacin (OFL), adriamycin hydrochloride (ADMh), tigecycline (TIC), minocycline (MCh), chloramphenicol (CAP), oxytetracycline (OTC) and tetracycline (TC), Table S1: The color coordinates of CIE chromaticity diagram of SiCDs@mMIP-cit-Eu probe for various concentrations of DPA (from 0 to 5.5 µM), Table S2: Detection performance comparison with other reported methods for TC sensing, Table S3: Detection performance comparison with other reported methods for OTC sensing, Table S4: Determination of TC in tap water, lake water, milk and honey samples by SiCDs@mMIP-cit-Eu probe.
