# Supplementary material for: Dual-Channel Probe of Carbon Dots Cooperating with Lanthanide Complex Employed for Simultaneously Distinguishing and Sequentially Detecting Tetracycline and Oxytetracycline
Source: Nanomaterials (Basel). 2021 Dec 31;12(1):128. doi: 10.3390/nano12010128 (PMC8747003; doi:10.3390/nano12010128)
Supplement: Supplementary file 1 [file nanomaterials-12-00128-s001.zip › nanomaterials-1519267-supplementary.pdf]

Supporting Information

Dual-Channel Probe of Carbon Dots Cooperating with Lanthanide Complex Employed for Simultaneously Distinguishing and Sequentially Detecting Tetracycline and Oxytetracycline

Lei Jia, Zhitao Xu, Rujie Chen, Xiangzhen Chen, Jun Xu*

College of Chemistry and Chemical Engineering, Henan Polytechnic University, No. 2001 Shiji Road, Jiaozuo, Henan, 454000, China

Corresponding Author: Jun Xu, E-mail: xjjl@hpu.edu.cn. Henan Polytechnic University, #2001 Shiji Street, Jiaozuo, Henan, 454000, China. Tel.: +86-391-3986812.

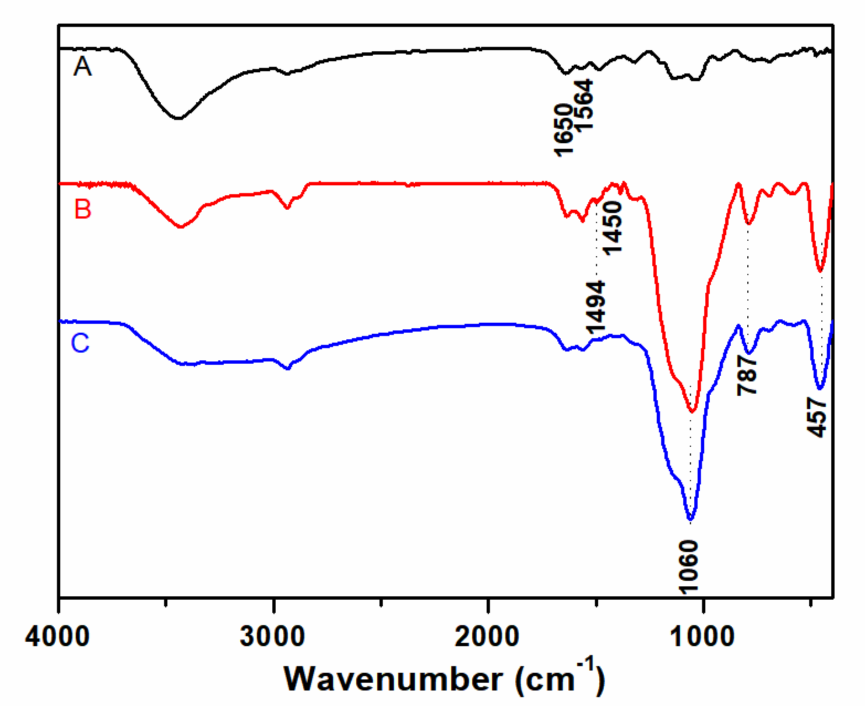

Figure S1 FT-IR spectrum of SiCDs (A), and SiCDs@mMIPs before (B) and after (C) solvent extraction of TC template molecule.

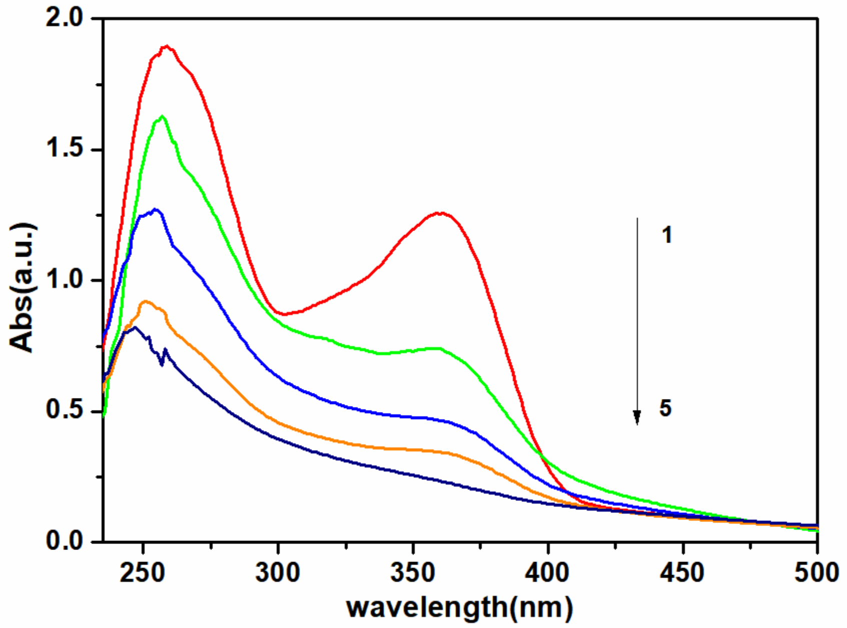

Figure S2 UV-vis absorption spectra of TC template molecularly imprinted SiCDs@mMIPs material after washing 5 times with acidified methanol.

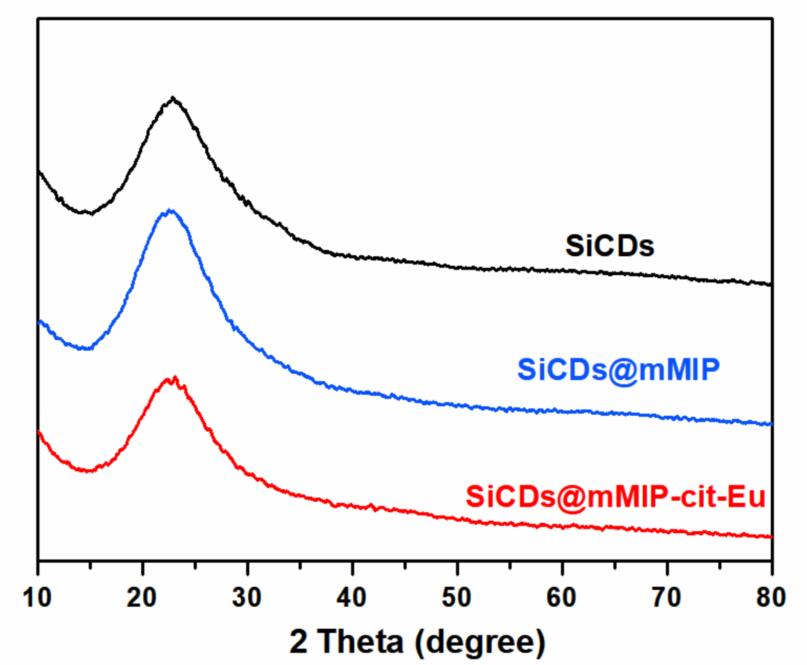

Figure S3 X-ray diffraction (XRD) patterns of SiCDs, SiCDs@mMIP,s and SiCDs@mMIPs-cit-Eu.

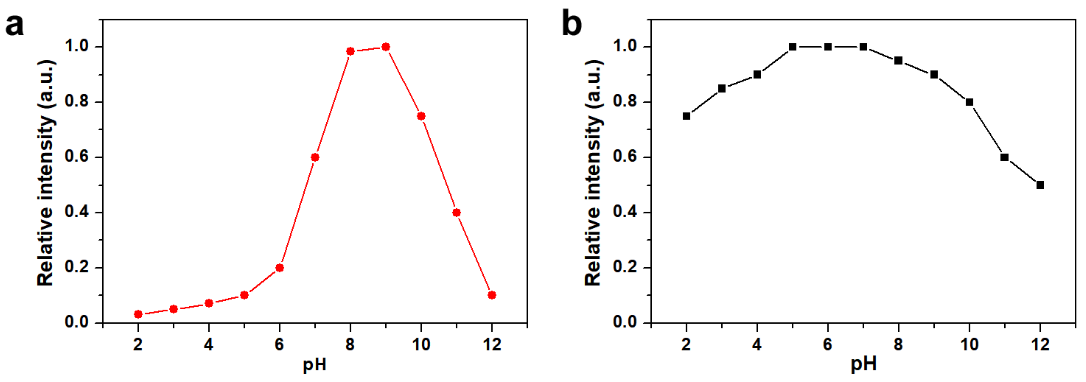

Figure S4 The effect of pH on fluorescence emission peak at 616 nm (a) and 450 nm (b).

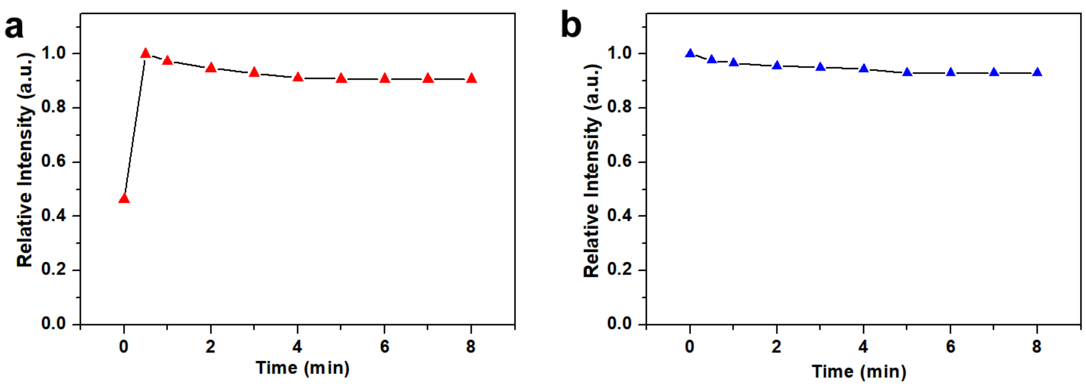

Figure S5 The response times of fluorescence emission peaks at 616 nm (a) and 450 nm (b) for TC detection.

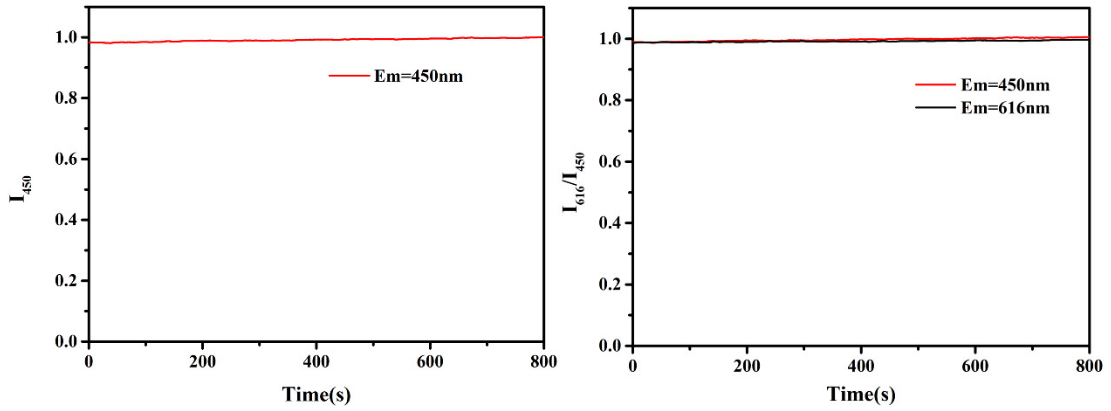

Figure S6 Fluorescence stability of SiCDs (left) and SiCDs@mMIPs-cit-Eu (right) after adding TC in 800 s.

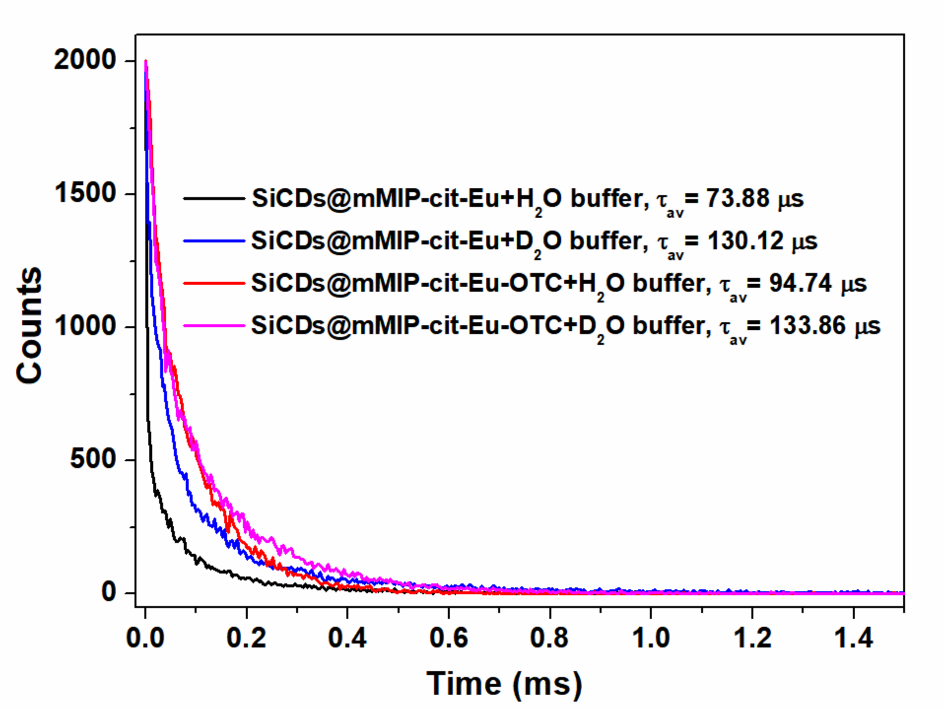

Figure S7 The lifetime of SiCDs@mMIP-cit-Eu and SiCDs@mMIP-cit-Eu-OTC nanomaterials in H_2O and D_2O buffers.

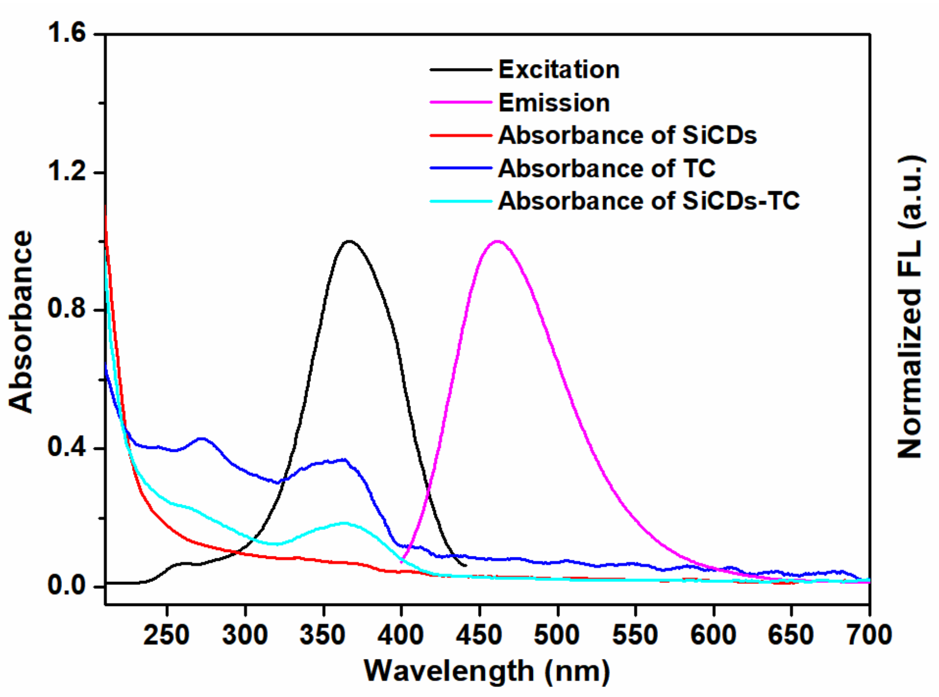

Figure S8 The excitation and emission spectra of SiCDs, and UV-vis absorption spectra of SiCDs, TC and SiCDs-TC.

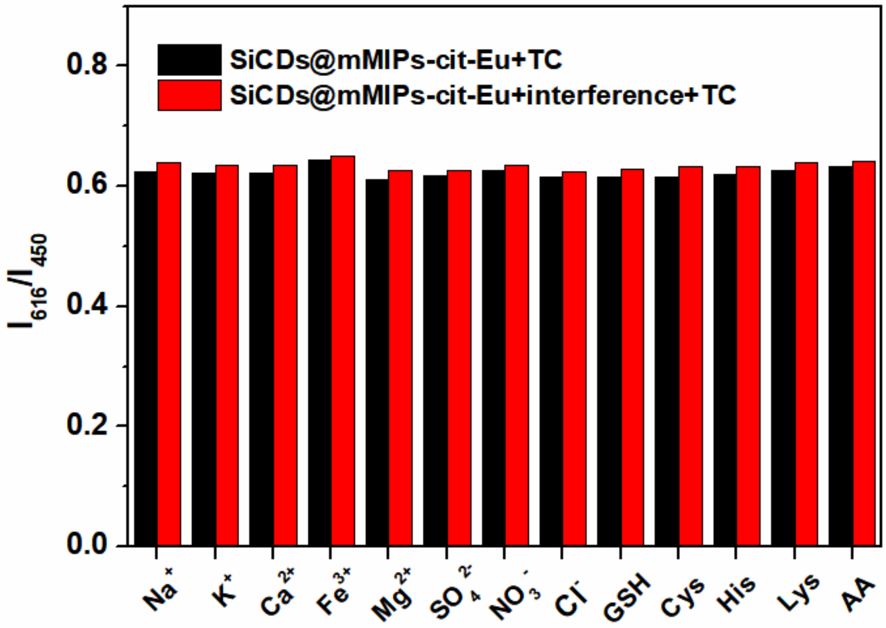

Figure S9 The influence of coexisting substances on the I_{616}/I_{450} of SiCDs@mMIP-cit-Eu in the presence of TC.

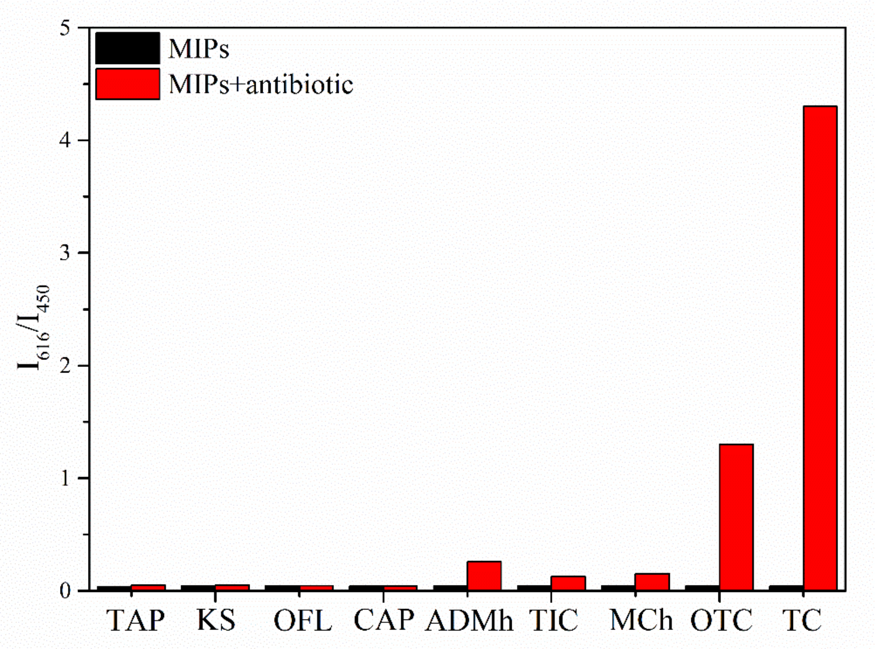

Figure S10 Fluorescence response (I_{616}/I_{450}) of SiCDs@mMIP-cit-Eu probe to thiamphenicol (TAP), kanamycin sulfate (KS), ofloxacin (OFL), adriamycin hydrochloride (ADMh), tigecycline (TIC), minocycline (MCh), chloramphenicol (CAP), oxytetracycline (OTC), and tetracycline (TC).

Table S1 The color coordinates of CIE chromaticity diagram of SiCDs@mMIP-cit-Eu probe for various concentrations of DPA (from 0 to 5.5 μM).

TC concentrations(μM)	0	0.1	0.2	0.3	0.4
x	0.184	0.187	0.192	0.198	0.200
y	0.188	0.188	0.192	0.190	0.190
TC concentrations (μM)	0.5	0.7	0.9	1.1	1.3
x	0.206	0.218	0.228	0.238	0.247
y	0.190	0.198	0.199	0.204	0.206
TC concentrations (μM)	1.5	2.5	3.5	4.5	5.5
x	0.260	0.312	0.377	0.412	0.445
y	0.210	0.225	0.245	0.258	0.268

Table S2. Detection performance comparison with other reported methods for TC sensing.

Methods	Detection range (μM)	LOD (μM)	Samples	Reference
Fe/Zn-MMT/GCE Electrochemistry	3-52	10	Feedstuff and meat	[1]
polymer inclusion membrane-HPLC method	1-100	0.23	Milk	[2]
Fe_3O_4 MNPs colorimetric sensor	0.1-1	0.045	Drugs	[3]
Europium-doped AgNP@SiO_2	0-6	0.083	Tap water	[4]
Nucleotide/lanthanide Coordination polymer	0.1-20	0.06	Milk	[5]
ZnO-CdS@Au	0.05-0.2	0.0045	Environmental water	[6]
SiCDs@mMIP-cit-Eu nanoprobe	0-5.5	0.005	Tap water, lake water, milk, honey,	This work

Table S3. Detection performance comparison with other reported methods for OTC sensing.

Methods	Detection range (μM)	LOD (μM)	Samples	Reference
N-CQDs	3.32-2.26	0.3739	River water, Tap water	[7]
SiNPs	0.2-20	0.18	Milk	[8]
Gold nanoclusters	0.375-2.5	0.15	human serum	[9]
Bodipy probe	0-42	0.72	Milk, honey, pork	[10]
WN-CQDs	0.25-100	0.077	Tap water, lake water, soil	[11]
CDs	0-40	0.41	water, milk, oil	[12]
SiCDs@mMIPs-cit-Eu	0-5.5	0.016	Tap water, lake water, Milk, honey	This work

Table S4 Determination of TC in tap water, lake water, milk, and honey samples by SiCDs@mMIP-cit-Eu probe.

Samples	Added (μM)	Determined (μM)	Recovery (%)	RSD (%)
Tap water	0	ND	-	-
	0.1	0.098±0.002	98.0	1.02
	1	0.997±0.02	99.69	1.70
	5	5.077±0.07	101.5	0.008
Lake water	0	ND	-	-
	0.1	0.104±0.001	104.3	2.70
	1	1.047±0.004	104.6	1.68
	5	4.937±0.08	98.7	0.076
milk	0	ND	-	-
	0.1	0.097±0.004	96.5	2.19
	1	1.033±0.01	103.3	1.75
	5	5.012±0.06	100.2	0.51
honey	0	ND	-	-
	0.1	0.102±0.003	102.0	2.05
	1	1.022±0.04	102.6	2.32
	5	4.891±0.07	97.8	0.73

References

[1] T. Gan, Z. Shi, J. Sun, Y. Liu, Simple and novel electrochemical sensor for the determination of tetracycline based on iron/zinc cations-exchanged montmorillonite catalyst, Talanta, 121(2014) 187-193.

[2] I. Perez-Silva, J.A. Rodriguez, M. Teresa Ramirez-Silva, M. Elena Paez-Hernandez, Determination of oxytetracycline in milk samples by polymer inclusion membrane separation coupled to high performance liquid chromatography, Analytica chimica acta, 718(2012) 42-46.

[3] Y. Wang, Y. Sun, H. Dai, P. Ni, S. Jiang, W. Lu, Z. Li, Z. Li, A colorimetric biosensor using Fe_3O_4 nanoparticles for highly sensitive and selective detection of tetracyclines, Sensors and Actuators B: Chemical 236 (2016) 621-626.

[4] P. Li, S. Kumar, K.S. Park, H.G. Park, Development of a rapid and simple tetracycline detection system based on metal-enhanced fluorescence by europium-doped AgNP@SiO_2 core–shell nanoparticles, RSC Advances 8 (2018) 24322-24327.

[5] H. Tan, C. Ma, Y. Song, F. Xu, S. Chen, L. Wang, Determination of tetracycline in milk by using nucleotide/lanthanide coordination polymer-based ternary complex, Biosens Bioelectron 50 (2013) 447-452.

[6] X. Zhang, R. Zhang, A. Yang, Q. Wang, R. Kong, F. Qu, Aptamer based photoelectrochemical determination of tetracycline using a spindle-like ZnO-CdS@Au nanocomposite, Microchimica Acta 184 (2017) 4367-4374.

[7] Qi. H, Teng. M, Liu. M, Liu. S, Li. J, Teng. C, Huang. Z, Liu. H, Shao. Q, U. Ahmad, Ding. T, Gao. Q, Guo. Z, Biomass-derived nitrogen-doped carbon quantum dots: highly selective fluorescent probe for detecting Fe^{3+} ions and tetracyclines, Journal of Colloid and Interface Science 539(2019) 332-341.

[8] Xu. N, Yuan. Y, Yin. J, Wang. X, Meng. L, One-pot hydrothermal synthesis of luminescent silicon-based nanoparticles for highly specific detection of oxytetracycline via ratiometric fluorescent strategy, RSC Advances 7(2017) 48429-48436.

[9] Xu. S, Li. X, Mao. Y, Gao. T, Feng. X, Luo. X, Novel dual ligand co-functionalized fluorescent gold nanoclusters as a versatile probe for sensitive analysis of Hg^{2+} and oxytetracycline, Analytical and Bioanalytical Chemistry 408(2016) 2955-2962.

[10] Xu. Z, Yi. X, Wu. Q, Zhu. Y, O. Minrui, Xu. X, First report on a BODIPY-based fluorescent probe for sensitive detection of oxytetracycline: application for the rapid determination of oxytetracycline in milk, honey and pork, RSC Advances 6(2016) 89288-89297.

[11] Gao. R, Wu. Z, Wang. L, Liu. J, Deng. Y, Fang. J, Liang. Y, Green preparation of fluorescent nitrogen-doped carbon quantum dots for sensitive detection of oxytetracycline in environmental samples, Nanomaterials 10(2020) 1561.

[12] Fu. Y, Huang. L, Zhao. S, Xing. X, Lan. M, Song. X, A carbon dot-based fluorometric probe for oxytetracycline detection utilizing a förster resonance energy transfer mechanism, Spectrochimica Acta Part A: Molecular and Biomolecular Spectroscopy 246(2021) 118947.
